# An Unusual Location of Juvenile Angiofibroma: A Case Report and Review of the Literature

**DOI:** 10.1155/2013/175326

**Published:** 2013-02-16

**Authors:** Ravishankar Pillenahalli Maheshwarappa, Amit Gupta, Juhi Bansal, Mahesh Virupaksha Kattimani, Sachin Shivayogappa Shabadi, Suresh Chandra Baser

**Affiliations:** Department of Radiodiagnosis, Ravindra Nath Tagore Medical College, Udaipur 313001, India

## Abstract

A 10-year-old boy presented with left-sided nasal obstruction and epistaxis. Endoscopic evaluation revealed a polypoid mass in the vestibule arising from the lateral wall of the nasal cavity anteroinferior to the left inferior turbinate. Computed tomography (CT) scan showed a soft tissue opacity in the vestibule of the left nasal cavity. After the endoscopic excision of the mass, postoperative and histopathological analyses confirmed the diagnosis of an angiofibroma.

## 1. Introduction 

Angiofibromas are highly vascular, nonencapsulated, and histologically benign but locally aggressive tumors which most commonly arise in the nasopharynx of adolescent males. It is a unique fibrovascular tumor with the specific histopathological finding of irregularly configured endothelial-lined vascular spaces embedded in a fibrous stroma. They usually arise from the posterolateral wall of the nasal cavity, where the sphenoidal process of the palatine bone meets the horizontal ala of the vomer and the pterygoid process. Angiofibromas constitute about 0.5% of all head and neck neoplasms [1]. These tumors may rarely localize in extranasopharyngeal sites. We report a case of angiofibroma in the left vestibule arising from the lateral wall of nasal cavity anteroinferior to the inferior turbinate.

## 2. Case Report 

**Figure 1:**
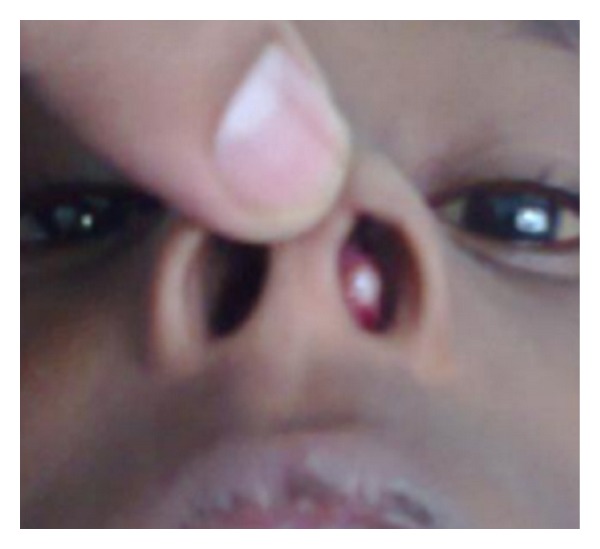
Reddish-gray-colored polypoidal mass in the left vestibule.

A 10-year-old boy came to the outpatient department with a four month history of progressive left-sided nasal obstruction and intermittent epistaxis. He had history of fever 5 days back. The patient had no other health problems. The coagulation status was normal. Anterior rhinoscopy revealed a fragile, lobular, red-grayish-colored, smooth, and polypoidal mass, arising anteroinferiorly to the left inferior turbinate filling the vestibule (Figure 1). Posterior rhinoscopy was normal in appearance.

**Figure 2:**
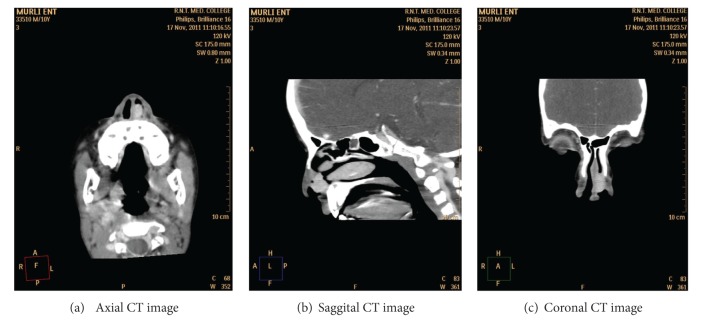
Axial, sagittal, and coronal CT images showing an intensely enhancing homogenous mass lesion in the left vestibule.

Computed tomography (CT) scan of the nose and paranasal sinuses demonstrated a soft tissue opacity that filled the vestibule of the left nasal cavity, without any sinus invasion and bony destruction. On postcontrast study, the soft tissue is showing intense enhancement (Figures 2(a), 2(b), and 2(c)).

The mass was later excised under general anaesthesia. It was lobular, smooth, red-grayish, about 12 mm long tumour, and with the diameter up to 8 mm. This was followed by profuse bleeding which was controlled with anterior intranasal packing. The mass was sent for histopathological analysis. The antibiotic therapy was used for ten days. The anterior nasal pack was removed on the fifth postoperative day and endoscopic nasal finding was good.

**Figure 3:**
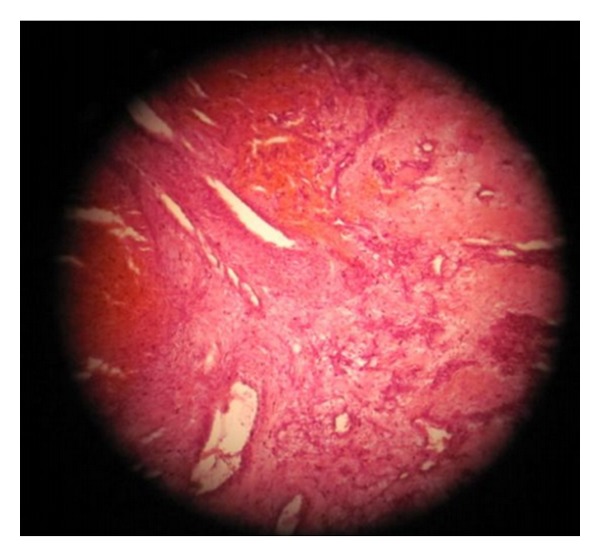
Histopathology showing irregular blood vessels ranging from capillaries and sinusoids to large vessels lined with one layer of flat endothelial cells lying in a fibrous stroma.

Histopathological examination of the excised masses (hematoxylin-eosin stain) showed a metaplastic squamous epithelium with the respiratory epithelium remnants on the tumor surface. Under the epithelium, we found many irregular blood vessels ranging from capillaries and sinusoids to large vessels, lined with one layer of flat endothelial cells lying in a fibrous stroma. Several fields of proliferative fibrosis at the sites of repetitive haemorrhages were seen (Figure 3). This suggested the diagnosis of angiofibroma.

## 3. Discussion 

Angiofibromas of the head and neck are benign nonencapsulated lesions occurring predominantly in the nasopharynx [1]. The tumour usually originates near the sphenopalatine foramen region, later it grows in all directions through multiple projection [2]. It has tendency to grow through high resistance ways between the bones, instead of only occupying space. Some authors believe that the tumor aggressiveness is related to the patient's age, observing that both intracranial and infratemporal invasion usually occur in younger patients [3].

There are number of theories have been put forward to explain the etiopathogenesis of angiofibromas. They can be developmental, hormonal, and genetic, but none of them found general acceptance. Currently juvenile angiofibromas are believed to be as vascular malformations, arising from discontinuous vascular basal laminae, focal lack of pericytes, and pronounced irregularity of the smooth muscle layers [4]. Angiofibroma has been hypothesized as a testosterone-dependent tumor that arises from a fibrovascular nidus in the nasopharynx that lies dormant until the onset of puberty. Hence, the incidence is more in males. 

According to Brunner, these tumors originate in the tissue of the anterior margin of the atlas at the lower surface of the sphenoid bone, which is called fascia basalis. During its development, this tissue extends up to the posterior part of the vomer and the ethmoid bone. We suggested that our patient's tumor had arisen from the periosteum on the anteroinferior end of inferior turbinate. The presence of this tumor on the anterior portion of the inferior turbinate indicated that the origin may be from ectopic tissues located further away to its usual place [2]. 

Primary extranasopharyngeal angiofibromas have been reported sporadically in the literature. They most commonly originate from the maxillary sinus. Other rare sites reported are the ethmoid and sphenoid sinuses, nasal septum, middle turbinate, inferior turbinates, conjunctiva, molar and retromolar region, tonsil, and larynx [2, 5, 6]. Unlike nasopharyngeal angiofibromas, extranasopharyngeal angiofibromas occur more frequently in females at later age [1]. In our patient case, the tumor pedicle was anteroinferior to the inferior turbinate and filling the vestibule. However, we could not find any case of the angiofibroma of this kind in the literature in English. 

The most frequent symptoms of nasopharyngeal angiofibromas are unilateral nasal obstruction, epistaxis, facial deformities, and pain. Extranasopharyngeal angiofibromas symptoms depends on the site of their occurrence [1].

The management angiofibromas includes preoperative radiological examination and biopsy [7]. Computerized tomography, MRI, and arteriography are valuable diagnostic procedures in the evaluation of nasopharyngeal angiofibromas [8]. Selective arteriography clearly demonstrates vascular pattern and blood flow dynamics and allows preoperative selective embolization to reduce intraoperative bleeding. Computerized tomography is sufficient for diagnosis, because it clearly delineates and identifies the tumor. Endoscopic examination is warranted, but biopsy is advised for confirmation after proper homeostasis [7].

The various modalities for the treatment of angiofibromas include surgery, hormonal therapy, radiation, and systemic chemotherapy [1]. However, surgery remains the primary course of treatment. In our case, the size and the location of tumor permitted us to remove it completely, using a simple endoscopic surgical procedure, without previous selective arteriography and embolization [9]. 

The differential diagnosis includes fibrosed antrochoanal and ethmoidal polyp and other fibrovascular tumors, such as capillary hemangioma, hemangiopericytoma, and solitary fibrous tumor [10].

## 4. Conclusion 

Extranasopharyngeal angiofibroma arising from the nasal cavity is an extremely rare tumour. The exact cause is not known. It probably comes from an ectopic tissue. Radiological and endoscopic examination along with histopathological analysis is necessary for its diagnosis. Surgical excision of the mass is the treatment of choice.
